# The Type 9 Secretion System Is Required for *Flavobacterium johnsoniae* Biofilm Formation

**DOI:** 10.3389/fmicb.2021.660887

**Published:** 2021-09-02

**Authors:** Todd J. Eckroat, Camillus Greguske, David W. Hunnicutt

**Affiliations:** ^1^School of Science, Penn State Erie, The Behrend College, Erie, PA, United States; ^2^Division of Natural Science, St. Norbert College, De Pere, WI, United States

**Keywords:** *Flavobacterium*, biofilm, motility, type IX secretion system, johnsoniae

## Abstract

*Flavobacterium johnsoniae* forms biofilms in low nutrient conditions. Protein secretion and cell motility may have roles in biofilm formation. The *F. johnsoniae* type IX secretion system (T9SS) is important for both secretion and motility. To determine the roles of each process in biofilm formation, mutants defective in secretion, in motility, or in both processes were tested for their effects on biofilm production using a crystal violet microplate assay. All mutants that lacked both motility and T9SS-mediated secretion failed to produce biofilms. A *porV* deletion mutant, which was severely defective for secretion, but was competent for motility, also produced negligible biofilm. In contrast, mutants that retained secretion but had defects in gliding formed biofilms. An *sprB* mutant that is severely but incompletely defective in gliding motility but retains a fully functional T9SS was similar to the wild type in biofilm formation. Mutants with truncations of the *gldJ* gene that compromise motility but not secretion showed partial reduction in biofilm formation compared to wild type. Unlike the *sprB* mutant, these *gldJ* truncation mutants were essentially nonmotile. The results show that a functional T9SS is required for biofilm formation. Gliding motility, while not required for biofilm formation, also appears to contribute to formation of a robust biofilm.

## Introduction

While bacteria are commonly studied in planktonic cultures, growing evidence suggests that association with surfaces may be more common than planktonic growth (O’Toole and Wong, 2016). Cells growing in biofilms produce more extracellular polysaccharide, exhibit lower growth rates, and have different gene expression profiles than cells growing planktonically (Donlan, 2002; Lo et al., 2009). There is a growing body of research on surface associated bacteria. This research provides insight into the genetic basis of biofilm formation and the physiology of cells associated with surfaces, especially within the Firmicutes (Blehert et al., 2003; Beenken et al., 2004; Branda et al., 2004) and *Proteobacteria* (O’Toole and Wong, 2016). Gliding bacteria are common on surfaces, so it is important to examine biofilm formation in these bacteria.

A unique system of gliding motility is found in many members of the phylum Bacteroidetes. Several recent studies illustrate the importance of biofilm formation in these bacteria. Basson et al. (2008) conducted studies on biofilm formation by *Flavobacterium johnsoniae*-like isolates from aquaculture facilities in 2008. The fish pathogen *F. psychrophilum*, for example, forms biofilms and these appear to correlate with virulence and antimicrobial resistance (Sundell and Wiklund, 2011; Cai et al., 2013; Levipan and Avendaño-Herrera, 2017). Biofilm formation in *F. psychrophilum* is reduced in type IX secretion system (T9SS) mutants (Barbier et al., 2020). Similar results have been observed for *Capnocytophaga ochracea* (Kita et al., 2016). Surface adhesion, an important aspect of biofilm formation, has also been reported to be dependent on T9SS components (Chen et al., 2019; Barbier et al., 2020).

*Flavobacterium johnsoniae* is one of the best-studied members of the *Flavobacterium* genus. It is capable of gliding motility and has the capacity to degrade polysaccharides, including chitin, dextran, pullulan, laminarin, pectin, and plant hemicelluloses such as xylan and xyloglucan (McBride et al., 2009). Due to the relative ease of maintaining it in the lab and a well-developed system for genetic manipulation, *F. johnsoniae* is a good model organism to study the basic biology of *Bacteroidetes*, including gliding motility and the T9SS.

Gliding motility of *F. johnsoniae* has been extensively studied since the 1970s (McBride, 2019). Numerous genes have been shown to be required for gliding motility. Gliding motility in *F. johnsoniae* involves the movement of an adhesin along helical tracks on the cell surface driven by proton motive force (Pate and Chang, 1979; Nakane et al., 2013; Shrivastava et al., 2015). Numerous mutants are available with disruption in genes coding for components of the motility system (McBride, 2019). Gliding proteins GldA, GldB, GldD, GldF, GldG, GldH, GldI, and GldJ are all required for gliding (Agarwal et al., 1997; Hunnicutt and McBride, 2000; Hunnicutt et al., 2002; McBride et al., 2003; McBride and Braun, 2004; Braun and McBride, 2005). The motility adhesin SprB is propelled by the gliding motor, along helical tracks on the cell surface, resulting in cell movement. *Flavobacterium johnsoniae* has other motility adhesins, such as RemA, that perform the same function on different surfaces (Rhodes et al., 2011; Nakane et al., 2013). SprB and RemA are dependent on the T9SS to reach the cell surface, and loss of T9SS function thus impacts gliding (Shrivastava et al., 2013). The T9SS and the gliding motility apparatus also appear to share some components. GldL and GldM, for example, are thought to power both secretion by the T9SS and movement of motility adhesins such as SprB along the cell surface (McBride and Nakane, 2015; Shrivastava et al., 2015; Hennell James et al., 2021).

Some components of the motility system are also used by the T9SS (McBride, 2019). In *F. johnsoniae*, the T9SS is composed of the gliding proteins GldK, GldL, GldM, GldN, SprA, SprE, SprF, and SprT. Proteins not associated with gliding (Plug, PorV, and PorU) are also involved in secretion (McBride, 2019). In part due to the intimate connection between gliding motility and the T9SS, determining whether motility or secretion is responsible for specific phenotypes has proven challenging. For example, it was shown that deletion of *gldJ* results in apparent instability of GldK protein (Shrivastava et al., 2013). This has complicated the determination of whether GldJ is involved in gliding, in secretion, or in both processes.

Importantly, several mutations were identified that separate gliding from secretion. *Flavobacterium johnsoniae porV* mutants, for example, retain motility but have a profound defect in secretion (Kharade and McBride, 2015). In contrast, loss of the major motility adhesin SprB results in a severe but incomplete defect in gliding, but has no effect on functioning of the T9SS (Nelson et al., 2008; McBride, 2019). More recently, Johnston et al. (2018) generated mutants that produce truncated GldJ proteins; these mutants were essentially nonmotile, but were competent for secretion. Here, we examined *F. johnsoniae* mutants that were defective for gliding motility, secretion by the T9SS, or both, to determine the roles of gliding and secretion in biofilm formation.

## Materials and Methods

### Bacterial Strains and Media

Strains used in this study are listed in Table 1. Casitone-yeast extract (CYE) broth (McBride and Baker, 1996) was made by mixing 10g casitone, 5g yeast extract, 1g MgSO_4_·7H_2_O, 10ml 1M TRIS, and 1L dH_2_O. The final pH of the broth was approximately 7.3. For experiments evaluating media concentration, CYE broth was diluted with sterile water to make the reduced nutrient media at 1/20, 1/40, 1/80, 1/160, and 1/320 the original concentration. CYE agar was made by adding 15g of agar per 1L of CYE broth prior to sterilization.

**Table 1:** *Flavobacterium johnsoniae* strains and plasmids used in this study.

Strain	Genotype or description	References
ATCC 17061T (UW101)	Wild type	McBride and Braun, 2004; Braun and McBride, 2005; McBride et al., 2009
ATCC 17061T (FJ1)	Wild type	McBride and Braun, 2004; Braun et al., 2005
UW 102-146	*gldA* null mutant	Agarwal et al., 1997
UW 102-103	*gldB* null mutant	Hunnicutt and McBride, 2000
UW 102-97	*gldD* null mutant	Hunnicutt and McBride, 2001
UW 102-77	*gldF* null mutant	Hunnicutt et al., 2002
UW 102-75	*gldG* null mutant	Hunnicutt et al., 2002
UW 102-52	*gldH* null mutant	McBride et al., 2003
UW 102-21	*gldJ* null mutant	Braun and McBride, 2005
UW 102-33	*gldN* null mutant	Rhodes et al., 2010
UW 102-41	*gldI* null mutant	McBride and Braun, 2004
UW 102-55	*gldJ* null mutant	Braun and McBride, 2005
CJ1432	Complement of *gldJ* mutant UW102-55 with pMM313	Braun and McBride, 2005
CJ1171	Complement of *gldI* mutant UW102-41 with pMM291	McBride and Braun, 2004
CJ1631A	*gldNO* deletion mutant	Rhodes et al., 2010
CJ1640	Complement of *gldNO* mutant CJ1631A with pTB79	Rhodes et al., 2010
CJ1922	*sprB* mutant	Nelson et al., 2008
CJ2405	Complement of *sprB* mutant CJ1922 with pSN60	Nelson et al., 2008
CJ2130	*porV* mutant	Kharade and McBride, 2015
CJ2169	Complement of *porV* mutant CJ2130 with pSSK03	Kharade and McBride, 2015
CJ2386	gldJ548 *rpsL2*: *gldJ* truncation after amino acid 548	Johnston et al., 2018
CJ2405	Complement of *gldJ* truncation mutant CJ2386 with pMM313	Johnston et al., 2018
CJ2443	gldJ553 *rpsL2*: *gldJ* truncation after amino acid 553	Johnston et al., 2018
CJ2484	Complement of *gldJ* truncation mutant CJ2443 with pMM313	Johnston et al., 2018
Plasmids		
pMM291	pCP11 carrying *gldI*; Ap^r^ (Em^r^)	McBride and Braun, 2004
pSN60	pCP29 carrying *sprB*; Ap^r^ (Cf^r^ Em^r^)	Nelson et al., 2008
pSSK03	pCP29 carrying *porV*; Ap^r^ (Cf^r^ Em^r^)	Kharade and McBride, 2015
pMM313	pCP11 carrying *gldJ*; Ap^r^ (Em^r^)	Braun and McBride, 2005
pTB79	*gldN* in pCP23; Apr (Tc^r^)	Braun et al., 2005

### Growth and Biofilm Assay

Growth and biofilm formation was quantified in flat-bottom, 96-well polystyrene plates (Corning Costar #3591, #3370, and #3396), using a modification of a standard crystal violet staining procedure (O’Toole and Kolter, 1998). Briefly, a 96-well plate containing 100μl of CYE broth of the appropriate concentration (undiluted, 1/20, 1/40, 1/80, 1/160, or 1/320 to evaluate effect of media concentration; 1/40 CYE for all subsequent experiments) in each well was inoculated with 2μl of overnight *F. johnsoniae* cultures in quadruplicate. Negative control wells contained CYE broth but were left uninoculated. The plate was incubated for the desired time (8, 16, or 67h to evaluate effect of time; 24h for all subsequent experiments) at 25°C. The plate was then scanned in a Molecular Devices Spectra Max M3 plate reader to determine optical density (OD) at 630nm. The cultures were aspirated out of the plate, and each well was washed six times with 150μl of dH_2_O. The wells were then stained with 100μl of 0.1% crystal violet and incubated at room temperature for 45min. After incubation, the crystal violet was removed, and the wells were washed six more times with 150μl of dH_2_O. The crystal violet remaining was then solubilized with 100μl of 98% EtOH and OD 630nm was recorded. Results are reported as relative OD values at 630nm (averages of at least four replicates; normalized to highest average in dataset or the wild type). Error bars are reported as 95% CI in the average OD. Statistical differences between groups were determined with GraphPad Prism 9.2.0 using one-way ANOVA followed by *post-hoc* Tukey test with a value of *p* of 0.05. Significance groups are labeled with letters above the error bars in alphabetical order starting from letter “a,” which was arbitrarily assigned the highest relative OD (Qais et al., 2021).

### Imaging

Cells were grown in 100μl of 1/40 CYE inoculated with 2μl of overnight *F. johnsoniae* culture. The media were contained in wells formed by placing silicone gaskets (Electron Microscopy Sciences), disinfected with 70% EtOH on sterile polystyrene cell culture slides (Nalge Nunc Instruments), and incubated overnight at 25°C in humid chamber. The cultures were removed and the wells carefully washed 3X with 100ml sterile dH2O, after which the gaskets were removed. Biofilms were visualized by placing a drop of ProLong Gold with DAPI (Molecular Probes/Life Technologies) and a glass coverslip on the slides and viewed on an Olympus Fluoview FV1200 inverted confocal laser scanning microscope using a 60X objective, 405nm excitation wavelength, and 461nm emission wavelength.

## Results

### Effect of Media Concentration and Incubation Time on Biofilm Formation

**Figure 1:**
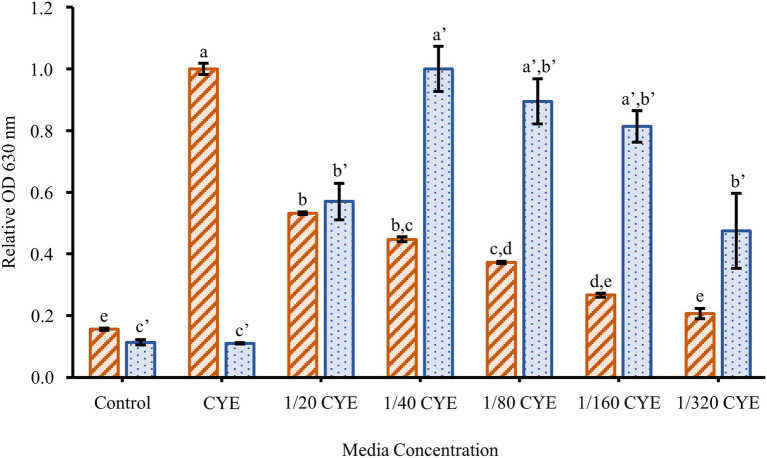
Effect of media concentration on biofilm formation. The planktonic growth (orange-striped bars) and biofilm formation (blue-dotted bars) of *Flavobacterium johnsoniae* wild type grown for 24h in a series of dilutions of casitone-yeast extract (CYE) media. Results are reported as relative optical density (OD) values (averages of at least four replicates; normalized to highest average in dataset). Error bars indicate 95% CI. Different letters above the error bars represent different significance groups among growth and biofilm measurements (one-way ANOVA followed by Tukey test, value of *p*=0.05).

**Figure 2:**
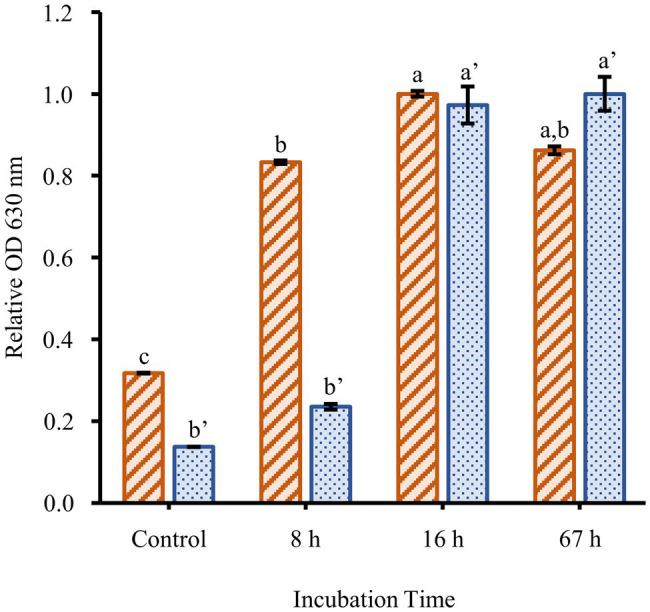
Time course of biofilm formation. The planktonic growth (orange-striped bars) and biofilm formation (blue-dotted bars) of *F. johnsoniae* wild type grown in a 1/40 dilution of CYE media. Results are reported as relative OD values (averages of at least four replicates; normalized to highest average in dataset). Error bars indicate 95% CI. Different letters above the error bars represent different significance groups among growth and biofilm measurements (one-way ANOVA followed by Tukey test, value of *p*=0.05).

To determine the effect of culture conditions on *F. johnsoniae* biofilm formation, cells were grown in full strength CYE and CYE diluted 1/20, 1/40, 1/80, 1/160, and 1/320 (Figure 1). Planktonic growth was highest in undiluted CYE, but the cells formed little or no biofilm under this condition. Biofilm formation increased with increasing media dilution while planktonic growth decreased. The most robust biofilm was observed in 1/40 CYE (Figure 1), and for this reason, a 1/40 dilution of CYE was used in all subsequent experiments. Biofilm formation was minimal at 8h incubation time, complete by 16h incubation time, and largely unchanged out to at least 67h (Figure 2).

### Effect of Motility and Secretion on Biofilm Formation

**Figure 3:**
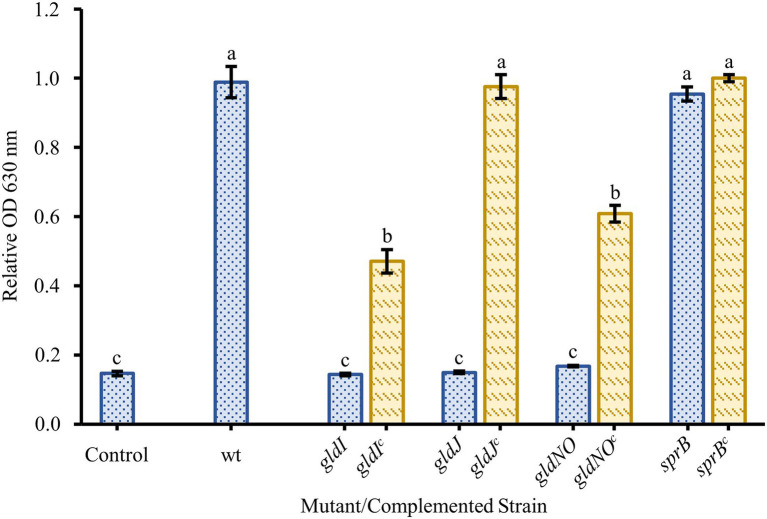
Effect of motility and type IX secretion system (T9SS) gene deletions on biofilm formation. Biofilm formation was determined for strains with knockouts in genes linked to gliding motility (*gldI*, *gldJ*), the T9SS (*gldJ*, *gldNO*), and colony spreading (*sprB*). Knockouts (blue-dotted bars) and complemented knockouts (yellow-dashed bars) of *F. johnsoniae* were *gldI* and *gldI^c^*: UW102-41 and CJ1171; *gldJ* and *gldJ^c^*: UW102-55 and CJ1111; *gldNO* and *gldNO^c^*: CJ1631A and CJ1640; and *sprB* and *sprB^c^*: CJ1922 and CJ2405. Results are reported as relative OD values (averages of at least four replicates; normalized to the wild type). Error bars indicate 95% CI. Different letters above the error bars represent different significance groups (one-way ANOVA followed by Tukey test, value of *p*=0.05).

**Figure 4:**
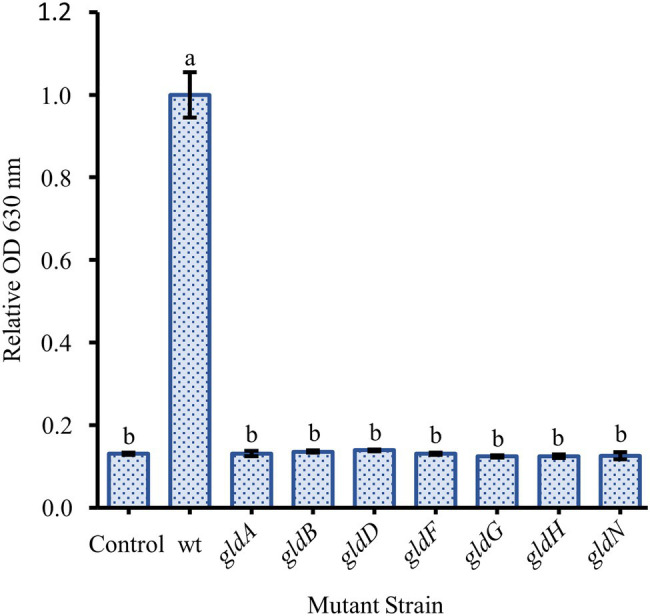
Effect of loss of motility on biofilm formation is consistent across many specific gene knockouts. Biofilm formation was determined for strains with knockouts in genes originally identified by the loss of gliding motility. The specific loss of function mutants were: *gldA*: UW102-146; *gldB*: UW102-103; *gldD*: UW102-97; *gldF*: UW102-77; *gldG*: UW102-75; *gldH*: UW102-52; and *gldN*: UW102-33. Results are reported as relative OD values (averages of at least four replicates; normalized to the wild type). Error bars indicate 95% CI. Different letters above the error bars represent different significance groups (one-way ANOVA followed by Tukey test, value of *p*=0.05).

Two nonmotile mutant strains of *F. johnsoniae* (UW102-41 and UW102-55) with point mutations in different motility genes (*gldI* and *gldJ*, respectively) were initially tested for biofilm formation. Neither formed biofilms (Figure 3). When the mutations in the motility apparatus were complemented (strains CJ1171 and CJ1111), motility and biofilm formation were restored. Biofilm formation in *gldI* and *gldJ* complemented mutants returned to roughly 50 and 100% of wild type, respectively (Figure 3). An additional mutant generated by unmarked deletion of *gldNO* exhibited the same phenotype. Conversely, an *sprB* deletion mutant produced robust biofilm, equal to wild type cells. Seven additional non-motile strains, each with a mutation in a different gene identified by a lack of gliding motility (*gldA*, *gldB*, *gldD*, *gldF*, *gldG*, *gldH*, and *gldN*), were evaluated for biofilm formation, and none formed biofilms (Figure 4).

**Figure 5:**
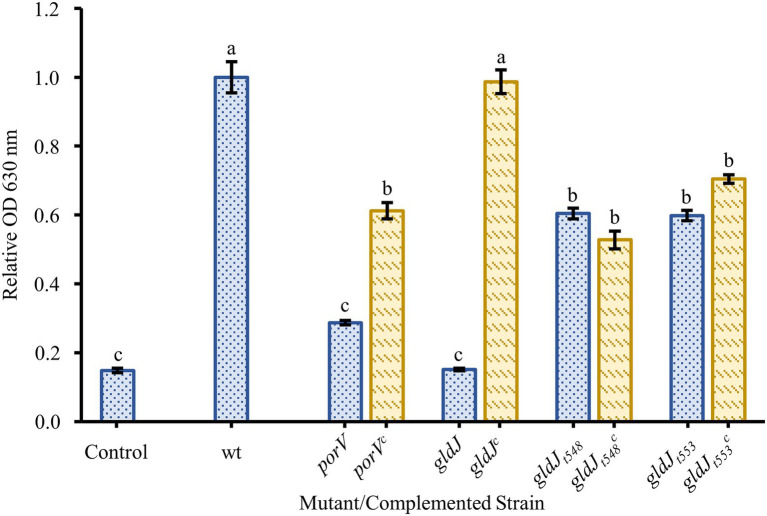
Biofilm formation by non-motile, T9SS-competent mutants and motile, T9SS-deficient mutants. Biofilm formation was determined for strains with knockouts in genes resulting in the loss of T9SS function (*porV*), motility (g*ldJ_t548_* and *gldJ_t553_*), and both T9SS and gliding motility (*gldJ*). Knockouts (blue-dotted bars) and complemented knockouts (yellow-dashed bars) of *F. johnsoniae* were *porV* and *porV^c^*: CJ2130 and CJ2169; *gldJ* and *gldJ^c^*: UW102-55 and CJ1111; *gldJ_t548_* and *gldJ_t548_^c^*: CJ2386 and CJ2405; and *gldJ_t553_* and *gldJ_t553_^c^*: CJ2443 and CJ2484. Results are reported as relative OD values (averages of at least four replicates; normalized to the wild type). Error bars indicate 95% CI. Different letters above the error bars represent different significance groups (one-way ANOVA followed by Tukey test, value of *p*=0.05).

Since recent findings demonstrated that the disruption of any of the motility genes tested above also impairs function of the T9SS, additional mutants were tested in an attempt to identify the relative contributions of motility and secretion. Strains with *porV* deletions, which are motile but profoundly secretion deficient and strains with *gldJ* truncations, which are secretion competent but non-motile, were assayed for biofilm formation (Figure 5). A strain with a deletion of the *porV* gene showed reduced biofilm formation, roughly 30% of wild type. Complementation of the *porV* mutant restored biofilm formation to roughly 60% of wild type. *Flavobacterium johnsoniae* strains CJ2386 and CJ2443 produce truncated GldJ proteins stopping after amino acids 548 and 553, respectively. These strains, missing the C-terminus of GldJ, are functional for T9SS-mediated secretion, but are nonmotile. These mutants with truncated GldJ proteins produced more robust biofilms than did the *porV* mutant, but had modest defects in biofilm production (roughly 60% of wild type). Complementation of these truncations with full-length *gldJ* on plasmid had little effect on biofilm formation, likely due to the high levels of expression of full length GldJ that can impair colony spreading and the incorporation of both truncated and full-length GldJ into the motility/secretion apparatus (Johnston et al., 2018).

**Figure 6:**
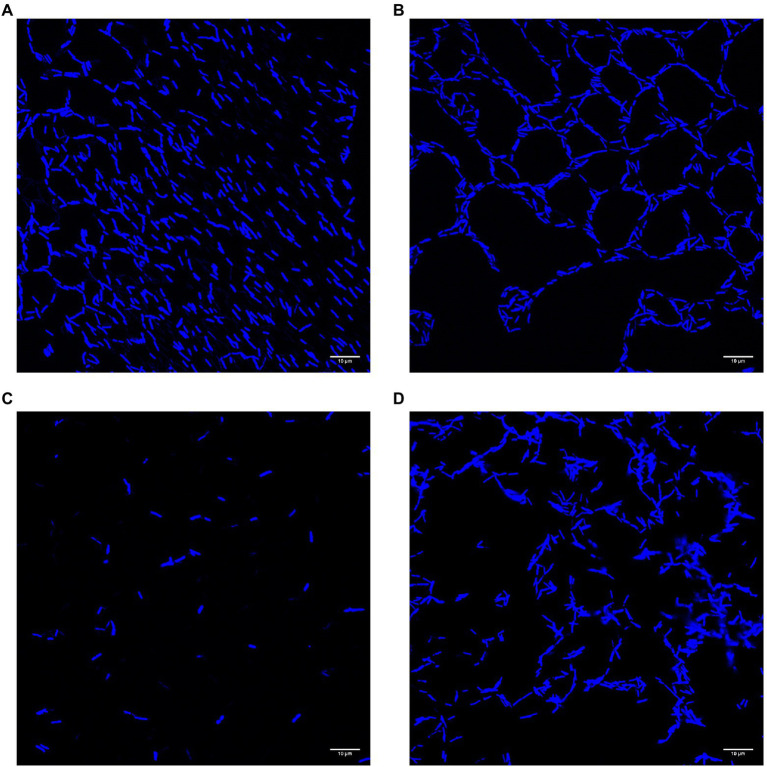
Confocal images of biofilms of wild type and deletions of *gldN* and *sprB* on polystyrene slides. Cells of biofilms grown in wells on polystyrene cell slides in 1/40 CYE overnight at 25°C in humid chamber were visualized by DAPI for confocal microscopy. **(A)** Wild type UW101 in a diffuse pattern. **(B)** Wild type UW101 in a “honeycomb” pattern. **(C)** *gldN* deletion CJ1631-A. **(D)** *sprB* deletion CJ1922.

Cells adhering to polystyrene slides and stained with DAPI showed variation in both cell density and arrangement (Figure 6). The cells of the wild type (UW101) were generally found either dispersed (A) or in a honeycomb pattern (B). Both conditions featured cells frequently arranged end to end. Cells of the *gldN* knockout (CJ 1631-A) were very sparse, reflecting the low levels of adherence measured by crystal violet staining (C). Cells of the *sprB* knockout (CJ 1922) were plentiful but showed less organization than the UW101 wild type (D). These images suggest that in addition to the quantitative differences between the biofilms measured in the crystal violet assay there are qualitative differences as well. These differences in cell arrangement warrant further study.

## Discussion

Surface association is a common feature of the vast majority of the Bacteroidetes. Many members of this phylum use insoluble nutrient sources and exhibit gliding motility, both of which require attachment and interaction with solid substrates rather than planktonic growth. Bacteroidetes are dominant components of biofilms in many environments (Edwards et al., 2010). *Capnocytophaga*, *Flavobacterium*, and *Bacteroides* biofilms have been studied in some detail (Basson et al., 2008; Cai et al., 2013; Kita et al., 2016; Levipan and Avendaño-Herrera, 2017). Many of these studies suggest the importance of genes involved in gliding motility and secretion in biofilm formation, which has also been found in other Gram-negative bacteria (Davey and O’Toole, 2000).

*Flavobacterium johnsoniae* has been a model system for studying genetics and physiology for decades. The genome sequence revealed unusual numbers of proteases and glycosidases, many of which appear to be linked to SucC/D-like systems, suggesting possible mechanisms for digesting large, insoluble macromolecules (Chen et al., 2009; McBride et al., 2009). The importance of both association with surfaces and secretion of digestive enzymes to these processes makes it important to gain understanding of *F. johnsoniae* biofilm formation. Given that the T9SS is a major system for the secretion of both soluble extracellular proteins and cell-surface proteins in many Bacteriodetes, it is not surprising that the T9SS is important in biofilm formation.

Many factors have been shown to affect biofilm formation in various bacteria. Since we suspected motility might play a role in *F. johnsoniae* biofilm formation, it was unsurprising that media dilution increased biofilm formation (Figure 1); low nutrient media have long been known to increase motility and colony spreading in this organism (Gorski et al., 1993). Motility has been shown to be important to biofilm formation in many organisms, and *F. johnsoniae* is no exception. All nonmotile mutants tested in our experiments exhibited essentially no biofilm formation. Strains with disruptions in *gldA*, *gldB*, *gldD*, *gldF*, *gldG*, *gldH*, *gldI*, and *gldJ* were indistinguishable from one another and the negative control in biofilm experiments (Figures 3, 4). Deletion of the T9SS component *gldNO* produced the same phenotype (Figure 3). The ability to form biofilms was restored by complementation in each mutant in which it was tested (*gldI*, *gldJ*, and *gldNO*). While initial interpretation of these data was that motility was required for biofilm formation, subsequent work in the McBride lab revealed that all of these mutants resulted in unstable g*ldK*, which also impairs secretion through the T9SS (Johnston et al., 2018). GldK is a core component of the T9SS (McBride, 2019).

Also of interest, deletion of the major gliding adhesin *sprB* did not reduce biofilm formation (Figure 3), indicating that the inability to secrete SprB was not the cause of impaired biofilm formation, resulting instead in biofilm formation at least as robust as wild type. Presumably other adhesins, such as RemA, are sufficient to allow biofilms to form. Individual cells adhering to polystyrene slides appeared to be less organized in the *sprB* deletion mutant, not as frequently aligning end to end as cells of the wild type (Figure 6). While deficient in colony spreading, *sprB* mutants retain a fully functional T9SS, and thus secrete RemA and dozens of other soluble and cell-surface proteins that may be important for biofilm formation (Nelson et al., 2008), or at least the adhesion of cells to polystyrene a measured by both the crystal violet assay and confocal microscopy.

In additional efforts to separate the effects of motility and secretion on biofilm formation, several newly available mutants were tested. *porV* deletion mutants fail to secrete most T9SS-secreted proteins, but they retain motility (Kharade and McBride, 2015). One likely reason for this is that among the few proteins that they secrete is the major motility adhesin, SprB (Kharade and McBride, 2015). Thus, *porV* mutants are motile but extremely limited in T9SS function, unable to secrete 26 of 33 proteins identified by Kharade and McBride (2015). The *porV* deletion mutant showed significantly reduced biofilm formation compared to wild type and *sprB* knockout, but slightly more than the knockouts of both motility and the T9SS such as *gldJ* (Figure 5). Thus, the loss of most T9SS function profoundly reduced the ability of *F. johnsoniae* to produce a biofilm, even though motility was retained.

To address the possibility that biofilm formation would be impaired by a loss of motility alone, two recently available mutants were tested. CJ2386, which carries a *gldJ* gene producing a GldJ protein truncated after amino acid 548, and CJ2443, which carries a *gldJ* gene producing a GldJ protein truncated after amino acid 553, are both secretion competent but profoundly deficient in motility. This portion of GldJ is sufficient to stabilize the core T9SS protein GldK in the membrane, allowing for full T9SS function. It does not, however, allow the essential function that full length GldJ plays in gliding motility. These *gldJ* truncation mutants secrete proteins through the T9SS, but they are effectively nonmotile. In our crystal violet biofilm assay, both GldJ truncations produced less robust biofilms than did the wild type, but their biofilms were far more robust than were those of any of the T9SS-deficient mutants (Figure 5). Taken together, these results suggest that both secretion and motility are involved in *F. johnsoniae* biofilm formation, but that secretion is more important to the formation of a robust biofilm formation than motility. Additionally, SprB does not seem to be needed for adherence to polystyrene but may be involved in biofilm organization.

## Data Availability Statement

The raw data supporting the conclusions of this article will be made available by the authors, without undue reservation.

## Author Contributions

TE, CG, and DH developed the modified crystal violet assay, performed the biofilm experiments, and conducted the data analysis. TE and DH produced the final figures and edited the manuscript. All authors contributed to the article and approved the submitted version.

## Conflict of Interest

The authors declare that the research was conducted in the absence of any commercial or financial relationships that could be construed as a potential conflict of interest.

## Publisher’s Note

All claims expressed in this article are solely those of the authors and do not necessarily represent those of their affiliated organizations, or those of the publisher, the editors and the reviewers. Any product that may be evaluated in this article, or claim that may be made by its manufacturer, is not guaranteed or endorsed by the publisher.
